# Hepcidin in hepatocellular carcinoma

**DOI:** 10.1038/s41416-022-01753-2

**Published:** 2022-03-09

**Authors:** Jonathan H. Joachim, Kosha J. Mehta

**Affiliations:** 1 GKT School of Medical Education, Faculty of Life Sciences and Medicine, King’s College London, London, UK; 2 Centre for Education, Faculty of Life Sciences and Medicine, King’s College London, London, UK

**Keywords:** Hepatocellular carcinoma, Cancer

## Abstract

Hepatocellular carcinoma (HCC) is one of the most common reasons for cancer-related deaths. Excess iron increases HCC risk. Inevitably, hepcidin, the iron hormone that maintains systemic iron homoeostasis is involved in HCC pathology. Distinct from other cancers that show high hepcidin expression, HCC patients can show low hepcidin levels. Thus, it is of immense clinical benefit to address the regulation and action of hepcidin in HCC as this may help in identifying molecular targets for diagnosis, prognosis, and therapeutics. Accordingly, this review explores hepcidin in HCC. It presents the levels of tissue and serum hepcidin and explains the mechanisms that contribute to hepcidin reduction in HCC. These include downregulation of *HAMP*, TfR2, HJV, ALK2 and circular RNA *circ_0004913*, upregulation of matriptase-2 and GDF15, inactivation of *RUNX3* and mutation in *TP53*. The enigmas around mir-122 and the functionalities of two major hepcidin inducers BMP6 and IL6 in relation to hepcidin in HCC are discussed. Effects of hepcidin downregulation are explained, specifically, increased cancer proliferation via activation of CDK1/STAT3 pathway and increased HCC risk due to reduction in a hepcidin-mediated protective effect against hepatic stellate cell activation. Hepcidin–ferroportin axis in HCC is addressed. Finally, the role of hepcidin in the diagnosis, prognosis and therapeutics of HCC is highlighted.

## Introduction

Hepatocellular carcinoma (HCC) represents approximately 90% of all primary liver cancers. It is an end-stage liver disease and is one of the most common reasons for cancer-related deaths [1]. Iron has an important role in cancer biology. The negative effects of iron overload on liver cells are well studied and have been linked to HCC development [2]. As such, excess iron increases cancer risk [3] and iron treatment to a HCC cell line has shown to increase mesenchymal and metastatic characteristics [4]. No wonder, patients with hereditary haemochromatosis (iron overload) show 20–200-fold increased risk of developing HCC [5].

Hepcidin (encoded by *HAMP* gene) is the liver-secreted 25-amino acid hormone that maintains systemic iron homoeostasis in the body. In HCC, iron-sensing is dysregulated [6], which in turn infers dysregulation of hepcidin and its modulators. In animal models, hepcidin deficiency increases the susceptibility for developing liver fibrosis, which is a risk factor for HCC. Also, hepatic hepcidin expression is reduced in cirrhosis [7], which is also a risk factor for HCC development. Collectively, this indicates an important role of hepcidin in the progression of liver pathology and in cancer development, progression, and metastasis.

Thus, it is extremely important to address hepcidin’s action and regulation in HCC. This might help identify molecular targets of pharmaceutical and clinical interest that may supplement and/or enhance the existing strategies of HCC diagnosis, prognosis, and therapeutics. As such, this approach has helped in the past. For example, understanding of HCC molecular mechanisms over the past decade has led to the development of newer treatment options such as tyrosine kinase inhibitors and immunotherapy [8].

Accordingly, this review investigates hepcidin in HCC. It presents the levels of serum and tissue hepcidin and explains the mechanisms that contribute to hepcidin reduction in HCC. The enigmas around the functionalities of the hepcidin inducers bone morphogenetic protein 6 (BMP6) and interleukin-6 (IL6), as well as miR-122 in relation to hepcidin levels in HCC, are discussed. Effects of hepcidin downregulation and the hepcidin–ferroportin axis in HCC are addressed. Finally, the role of hepcidin in the diagnosis, prognosis and therapeutics of HCC is highlighted.

## How hepcidin maintains systemic iron homoeostasis

**Fig. 1:**
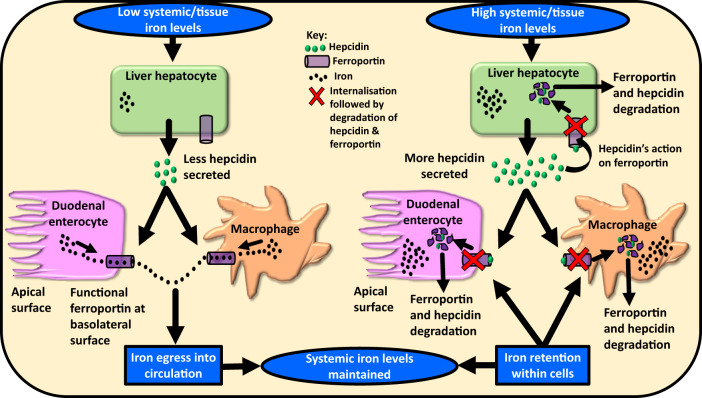
Hepcidin–ferroportin axis maintains systemic iron homoeostasis. The figure shows the three main cell types involved in maintaining systemic iron homoeostasis, namely, hepatocytes, macrophages, and enterocytes. Under iron-deficit conditions, hepcidin secretion by hepatocytes is reduced. This prevents ferroportin degradation and allows iron egress into the circulation. Under iron-excess conditions, hepcidin secretion by the hepatocytes increases. Hepcidin binds to ferroportin, and both hepcidin and ferroportin are degraded intracellularly. This prevents iron entry into the circulation and thereby systemic iron homoeostasis is restored.

As depicted in Fig. 1, upon elevation of iron in the circulation or in the liver, hepatic hepcidin expression increases and hepcidin is secreted into the circulation. Circulatory hepcidin binds to the sole known cellular iron-exporter ferroportin. Ferroportin is expressed in iron-storing and iron-transporting tissues, i.e. present on various cell types, including the iron-storing hepatocytes, iron-recycling macrophages in the spleen and liver, as well as the duodenal enterocytes. The binding of hepcidin to ferroportin causes internalisation and degradation of both hepcidin and ferroportin. This not only prevents iron egress into the circulation from the iron-storing and iron-recycling cells but also reduces dietary iron absorption via the duodenal enterocytes, thereby preventing further systemic iron elevation (Fig. 1).

In contrast, when systemic iron levels are low (e.g. during iron deficiency or haemorrhage) or when the erythropoietic demand is high, hepatic hepcidin expression is repressed. This prevents excessive ferroportin degradation on cell surfaces and allows cellular iron egress into the circulation, simultaneously promoting greater iron entry into the circulation (greater absorption of dietary iron) via the duodenal enterocytes (Fig. 1). The aim is to mobilise iron and provide it for red blood cell development (predominantly), and for various activities of other cell types. Thus, hepcidin regulates systemic iron levels [9, 10].

## Physiological modulators of hepcidin expression

There are three main physiological modulators of hepatic hepcidin expression. (i) systemic and tissue iron levels [involving diferric transferrin, BMPs and BMP-SMAD pathway], (ii) infection/inflammation (involving IL6) and (iii) erythropoiesis/hypoxia (involving erythropoietin-induced erythroferrone and hypoxia-inducible factors).

High tissue/systemic iron levels and inflammation induce hepcidin expression whereas iron deficiency and high erythropoietic demand suppress hepcidin expression [10, 11]. Hypoxia (particularly through hepatic hypoxia-inducible factor 2 alpha) suppresses hepcidin expression via an erythropoietin-facilitated increase in erythropoiesis [12, 13]. Indeed, there are other regulators of hepcidin such as growth factors like hepatocyte growth factor and epidermal growth factor that suppress hepcidin induction, sex hormones like progesterone and testosterone, where the latter can reduce hepcidin expression, and erythroid regulators like growth/differentiation factor-15 (GDF-15) and twisted gastrulation BMP signalling modulator-1 (TWSG1) that have shown to suppress hepcidin expression in primary human hepatocytes [14].

## Serum and tissue (liver) expression of hepcidin in HCC

Although other tissues secrete small amounts of hepcidin, the main source of hepcidin that regulates systemic iron levels is the liver. Thus, hepcidin is predominantly secreted by the liver and HCC is cancer of the liver. Thus, hepcidin dysregulation in HCC is expected.

The normal range of serum hepcidin in the human body has been between 0.4 and 23.3 nM [15] and more specifically between 2 and 20 nM [10]. Table 1 shows the hepcidin levels in HCC samples compared to controls in those studies.

**Table 1:** Hepcidin levels in hepatocellular carcinoma.

Distinct from other cancers that show elevated serum hepcidin, HCC patients can show low levels of serum hepcidin [6, 16, 17]. Also, human HCC tissues have shown lower hepcidin expression than adjacent non-cancerous liver tissue or normal liver tissues [6, 16–20], animal models of HCC have shown low hepcidin expression [7, 21], and HCC cells lines have shown lower hepcidin expression compared with primary human hepatocytes [6] and human liver samples [22] (Table 1).

Contrasting results have been reported too. A study that analysed hepcidin expression using GEO dataset GSE57957 reported that hepcidin was upregulated in HCC, i.e. hepcidin expression in HCC tissues was higher than that in surrounding non-tumorous tissues [23]. Similarly, patients with hepatitis-B-virus (HBV)-induced HCC showed higher serum hepcidin levels compared with healthy controls [24] (Table 1). Another contrasting aspect is related to differences in hepcidin expression in the presence or absence of multiple HCC tumours. Kijima et al. reported that hepcidin expression did not significantly differ between tumours at varying levels of differentiation, the number of tumours or vessel invasion [17] whereas another study reported that hepcidin mRNA expression was much lower in patients with multiple tumour masses [25]. Kijima et al. reported that serum hepcidin-25 concentrations did not co-relate with hepcidin mRNA expression in cancerous or non-cancerous tissue. Essentially, while hepcidin mRNA expression was low, serum hepcidin levels in HCC were high in some patients and normal in others (Table 1) [17]. The reason for this needs to be investigated. Such discordance between mRNA and protein expression has been previously indicated for hepcidin [26, 27], ferroportin [28] and other proteins too [4].

## Mechanisms underlying hepcidin downregulation in HCC

### Enigmas around BMPs, IL6, microRNA-122 and hepcidin suppression in HCC

BMP6 and IL6 are two major stimulators of hepcidin induction in hepatocytes in response to tissue iron-loading and inflammation, respectively [10].

The BMPs act as ligands and activate the BMP-SMAD pathway, which is the main cell signalling pathway that regulates hepatic hepcidin expression in response to iron [14]. BMP6 is the most potent and predominant hepcidin inducer in response to excess iron but other BMPs like BMP9, BMP4 and BMP2 (BMP2 seems to stimulate basal hepcidin induction) can induce hepcidin transcription in HCC cell lines, primary human hepatocytes and mice models [29]. Interestingly, BMPs 6 and 4 are elevated in the liver of HCC patients [6, 30], BMP4 is strongly expressed in HCC tissues and is induced by hypoxia in HCC [30], and so is BMP9 [31]. Thus, it would be expected that high expression of these BMPs would elevate hepcidin expression in HCC. However, this contrasts with what is observed in HCC, which is mostly low hepcidin expression.

Likewise, another baffling element that defies the norm in the context of hepcidin in HCC is IL6. IL6 is the main mediator of acute-phase response and hepcidin is characterised as an acute-phase protein [32]. Normally, hepcidin is induced by inflammation, particularly IL6 via the JAK-STAT3 pathway and the non-canonical BMP pathway [11]. Increased serum IL6 levels correlate with increased risk of developing HCC and HCC patients show increased levels of serum IL6 [33]. Relatedly, in HCC, the JAK/STAT pathway is aberrantly activated, which promotes tumour proliferation, invasion and metastasis [34]. Thus, in HCC, IL6-mediated hepcidin upregulation would be expected. However, this contrasts with the observed low levels of hepcidin in HCC, which cannot be related to the excessive IL6 levels observed in HCC patients.

Yet another inexplicable element is microRNA miR-122 in HCC. miR-122 is liver specific and regulates hepcidin (*HAMP*) mRNA expression as well as tissue iron levels. In mice models, its inhibition leads to increment not only in *Hamp* expression but also other proteins that participate in *Hamp* induction, namely Hfe and Hjv [35]. miR-122 is reported to be specifically and significantly downregulated in HCC in animal models as well as humans [36]. Based on the function of miR-122, hepcidin expression should increase in HCC, but this is not the case, at least in the majority of HCC cases.

**Fig. 2:**
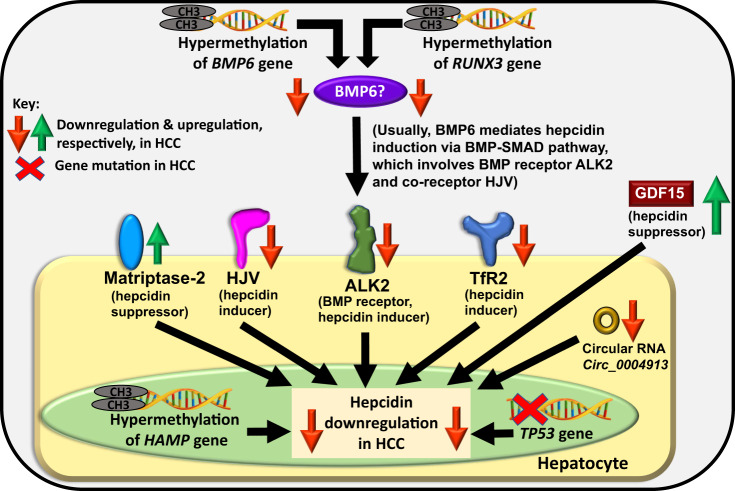
Plausible cellular mechanisms mediating hepcidin downregulation in hepatocellular carcinoma (HCC). The figure shows alterations in the hepcidin gene (*HAMP*) and changes in some hepcidin-modulatory proteins in HCC that may be fully or partly responsible for hepcidin downregulation in HCC. Note that BMP6 upregulation in HCC has been observed and this needs to be investigated.

Thus, in HCC, it appears that the usual hepcidin-uplifting effect of these hepcidin inducers is nullified/suppressed and other mechanisms and effectors take precedence. Some of these events and mechanisms are explained underneath and may explain the reason for hepcidin downregulation in HCC (Fig. 2).

### Hypermethylation of *BMP6*

BMP6 is the predominant ligand which when bound to its receptor (and co-receptors) on the hepatocyte cell surface induces hepcidin expression via the BMP-SMAD pathway in the hepatocytes in response to tissue iron excess [11]. Hypermethylation of *BMP6* that leads to its downregulation has been observed in HCC [20, 37]. Also, BMP6 was found to be downregulated when data were analysed from NCBI/GEO dataset GDS1385 that was generated from a rodent liver cancer model [20]. Thus, downregulated BMP6 may partly explain hepcidin reduction in HCC (Fig. 2). Note that BMP6 has been found to be upregulated in HCC, as discussed previously, so BMP6 in HCC needs to be further investigated.

### Downregulation of *HAMP* (hepcidin encoding gene)

One reason for low levels of hepcidin in HCC could be the downregulation of the hepcidin gene itself. In HCC tissues, increased methylation of the DNA on *HAMP* promoter region has been observed. This suppresses *HAMP* transcription [16], thereby producing less hepcidin in HCC (Fig. 2). Interestingly, such downregulation occurs despite normal serum iron (131.4 ± 23.4 mg/dL) and normal [179.5 (14–232.9 ng/mL)] or high [414.4 (328.2–1121 ng/mL)] ferritin levels in some HCC patients, as reported in one study [17] or elevated levels of iron, ferritin and transferrin saturation in the sera of HCC patients compared to control patients, as reported in another study [6].

### Downregulated TfR2 or HJV expression: dysregulation of iron-sensing in HCC

Transferrin receptor-2 (TfR2) is one of the iron-sensing proteins on the hepatocyte cell surface. It plays an essential role in sensing plasma iron levels and inducing hepcidin expression in the hepatocytes [38]. HCC patients show downregulated tissue expression of TfR2 compared with non-tumorous liver tissues [6, 18, 19]. Thus, TfR2 downregulation in HCC could be partly responsible for reduced hepcidin expression in some cases of HCC (Fig. 2).

Hemojuvelin (HJV) is a membrane-bound iron-sensing protein, highly expressed on the hepatocyte cell surface. It acts as a co-receptor to the BMP receptor and helps in forming the iron-sensing complex on the hepatocyte cell surface. It plays an essential role in iron-induced hepcidin expression via BMP-SMAD pathway [11]. In HCC, there is decreased mRNA stability of HJV, which remarkably reduces HJV in HCC cell lines and tissues. In turn, this reduces hepcidin induction [6] (Fig. 2).

Notably, a study reported no significant difference in HJV expression between HCC tissue and adjacent non-tumorous liver tissue [18]. Along similar lines, a study reported that TfR2 mRNA was not suppressed in HCC [17]. In such HCC cases, other hepcidin-suppressing mechanisms may be the cause of the observed hepcidin downregulation. Alternatively, such cases may explain the normal or high hepcidin levels found in selected cases of HCC. This needs to be further investigated.

### ALK2 downregulation: dysregulation of iron-sensing in HCC

To induce hepcidin, BMP6 binds to BMP type-1 receptors ALK2 or ALK3 (and BMP type-2 receptors) on the hepatocyte cell surface and activates the BMP-SMAD pathway. This highlights the importance of ALK2 in hepcidin induction. ALK2 is downregulated in HCC cell lines and HCC tissues [6], which could explain the reduced hepcidin expression in HCC (Fig. 2).

### Increased matriptase-2 expression

Matriptase-2 is a negative regulator of hepcidin, i.e. a suppressor of hepcidin induction. On the hepatocyte cell surface, it cleaves HJV, which is a part of the iron-sensing complex and a hepcidin inducer. Thereby, matriptase-2 suppresses hepcidin induction [11]. Considering some observations like matriptase expression (both protein and mRNA) is significantly higher in carcinoma cells compared with normal human prostate epithelial cells [39], tissue expression of matriptase is increased in the liver of HCC [40], and matriptase-2 is highly expressed in liver cancer cell line [41], it can be postulated that HCC patients may show high hepatic matriptase-2 expression. If so, then this would increase HJV cleavage from the hepatocyte cell surface causing downregulation of hepcidin expression or prevention of hepcidin upregulation in HCC (Fig. 2). Experiments need to be conducted to ascertain this. The Human Protein Atlas identifies matriptase-2 as a favourable prognostic marker for liver cancer.

### *RUNX3* inactivation

RUNX3 is a transcription factor that can act as a tumour suppressor and repress cancer cell migration and metastasis in HCC. It plays a role in iron biology too. It enhances the activity of *BMP6* promoter and allows *BMP6* upregulation in response to iron. Essentially, iron-induced elevation in BMP6 (and thereby, possibly BMP6-induced hepcidin induction) is mediated via RUNX3. Runx3 preserves Bmp signalling, regulates liver iron and it can prevent liver iron loading in mice [42]. In HCC tissues, *RUNX3* is hypermethylated leading to its inactivation and downregulation [43, 44]. This could have a knock-on effect on *BMP6* induction and activity. Since BMP6 is a hepcidin inducer, diminished BMP6 expression could lead to hepcidin repression in HCC, and thus, inactivated *RUNX3* could be one of the reasons for hepcidin suppression in HCC. Since RUNX3 has been shown as an independent prognostic factor for overall 5-year survival and disease-free survival in patients, it could be used for prognosis, and possibly as a therapeutic target for HCC [44].

### *TP53* mutation

p53 is a tumour suppressor protein (encoded by *TP53* gene) that is induced in stressful conditions like oncogene activation or DNA damage. It is regulated by alterations in iron metabolism whereby excess iron downregulates p53 expression [45]. *HAMP* promoter has a p53 response element. The binding of p53 to the *HAMP* promoter activates *HAMP* transcription whereas silencing of *TP53* decreases *HAMP* expression [46]. Essentially, *HAMP* transcription is activated by p53. Among the several genetic and epigenetic alterations that occur in liver cancer, *TP53 i*s frequently mutated in HCC [47, 48] and recurrent HCC [49]. Thus, hepcidin downregulation in HCC could be related to *TP53* mutation in HCC, as the data indicate a role of *TP53* silencing in decreasing *HAMP* expression in HCC [46] (Fig. 2).

### Increased GDF15 levels

GDF15 is usually induced under stressful conditions to maintain cell and tissue homoeostasis. In HCC, GDF15 has shown both tumour-promoting and anti-tumour effects [50]. It is overexpressed in liver cancer tissues [51] and serum levels of GDF15 are elevated in HCC patients [50]. Compared with healthy controls (serum GDF15 levels of 0.31 ± 0.01 ng/mL), HCC patients showed significantly increased GDF15 levels (6.66 ± 0.67 ng/mL) and so did the patients with cirrhosis (6.51 ± 1.47 ng/mL) [52]. GDF15 has been shown to suppress hepcidin expression in primary hepatocytes [11]. Assuming that GDF15 exhibits the same suppressive effect on hepatic hepcidin expression in humans, high GDF15 levels in HCC could downregulate hepcidin expression (Fig. 2).

### Downregulated circular RNA *circ_0004913*

The circular RNA *Circ_0004913* can regulate HCC progression. Expression of *circ_0004913* in HCC cells impedes cell proliferation, migration, and invasion, thereby showcasing tumour-suppressive features of this circular RNA. It targets microRNA-184, which in turn targets *HAMP*. *HAMP* expression positively correlates with *circ_0004913* expression, i.e. lower *circ_0004913* is linked with lower expression of *HAMP*. Notably, *Circ_0004913* is downregulated in HCC. Thus, this could be one of the reasons for hepcidin downregulation in HCC (Fig. 2) [53].

**Fig. 3:**
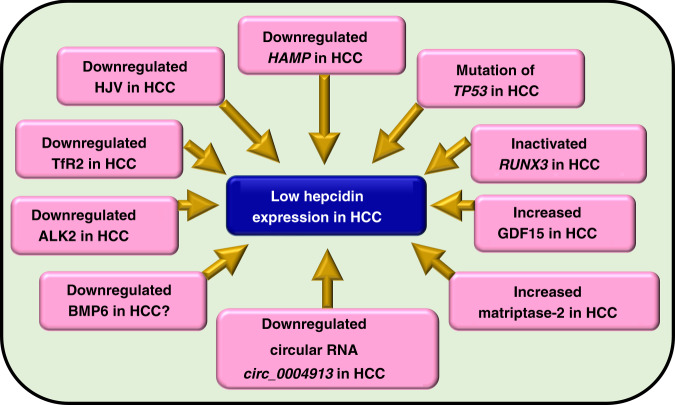
Summary of events underlying hepcidin suppression in hepatocellular carcinoma. The figure indicates plausible events in hepatocellular carcinoma (HCC) that may fully or partly, collectively or independently and to a greater or lesser extent be responsible for reduced hepcidin expression in HCC. The question mark indicates uncertainty around the expression of BMP6 in HCC as BMP6 is found to be raised in HCC, but BMP6 hypermethylation and thereby its downregulation has also been observed.

Plausible events underlying hepcidin downregulation in HCC have been summarised in Fig. 3.

### Is hepcidin downregulation due to cirrhosis or HCC?

Is the observed downregulation of hepcidin in HCC due to cirrhosis or due to HCC itself? This is an interesting question. Notably, there is an overlap between the different stages of the pathological spectrum of chronic liver disease. Generally, liver pathologies show an increasing pathological gradient of un-demarcated stages including hepatic steatosis (fatty liver), steatohepatitis (fatty liver and inflammation) or hepatitis (liver inflammation), and liver fibrosis followed by cirrhosis, which may lead to HCC. The pathological continuum makes it challenging to accurately dissect the stages and allocate a particular alteration (here hepcidin-related) exclusively to a specific pathological stage. For instance, while low level of hepcidin is observed in HCC patients [6, 16, 17], cirrhotic patients also show reduced hepcidin expression independent of disease aetiology [16, 54, 55], and hepcidin:ferritin ratio has been shown to decrease with fibrosis progression [15]. This collectively demonstrates decreased or decreasing hepcidin expression that spans across the stages of fibrosis, cirrhosis, and HCC. Therefore, it is likely that when fibrosis/cirrhosis progresses to HCC, it may ‘carry forward’ its downregulated hepcidin expression to the next stage of HCC, and this may contribute to the observed downregulation of hepcidin in HCC.

In that case, some of the hepcidin-suppressing mechanisms discussed here in the context of HCC may well begin at the cirrhosis stage. For example, while *TP53* mutations have been observed in HCC [47, 48], these mutations have been frequently found in patients with liver cirrhosis too. It is possible that *TP53* mutations at the cirrhotic stage may be one of the factors responsible for progression to HCC [56] as these mutations may well occur before HCC development [57]. Since p53 can activate *HAMP* transcription [46], *TP53* mutations at the cirrhosis stage may cause *HAMP* downregulation at this stage, and both *TP53* mutations and the downregulated hepcidin may prevail from there onwards up to the HCC stage. Another example is GDF15 in HCC. Increased GDF15 levels are observed in both cirrhosis and HCC stages [52] and these high levels at the cirrhotic stage may be ‘carried forward’ to the HCC stage that may contribute to the observed hepcidin downregulation in HCC. Collectively, these examples suggest that the observed downregulation of hepcidin in HCC and the mechanisms mediating its downregulation may well begin at a stage earlier than the HCC stage and may not be exclusive to the HCC stage.

On the other hand, it is possible that hepcidin increment in HCC may be exclusive to the HCC stage. For example, in a study, mean serum hepcidin levels in HBV-related cirrhosis patients did not significantly differ from the healthy controls while hepcidin levels in HBV-HCC patients were higher than controls (Table 1) [24]. This suggests that it is possible for hepcidin to increase at the HCC stage without being altered at the cirrhosis stage in some cases.

However, HCC can occur without cirrhosis [58–62]. In non-cirrhotic HCC, it is possible that one or some of the hepcidin-suppressing mechanisms discussed here (and more to be discovered in the future), including hypermethylation of *BMP6* and *RUNX3*, downregulations of TfR2, HJV, ALK2 and circular RNA *circ_0004913*, and increment in matriptase-2 may be exclusive to the HCC stage. This is a hypothesis and requires investigation.

## Effect of hepcidin downregulation on cancer proliferation and metastasis in HCC

Shen et al. showed that knockdown of *HAMP* in human liver carcinoma cell lines led to increased cell proliferation and increased migration ability, in addition to increased cellular iron levels. The former result was replicated in mice where groups with reduced *Hamp* expression showed higher tumour weights compared to controls [63]. The STAT3 pathway affects the cell cycle and iron plays an important role in inducing this pathway in HCC [64]. Shen et al. observed that in a human liver cancer cell line and in tumour tissue, *HAMP* downregulation caused increments in components of the cyclin-dependent kinase-1/STAT3 (CDK1/STAT3) pathway, namely, CDK1, STAT3 and phospho-STAT3 [63]. Thus, data showed that reduced *HAMP* expression could activate this pathway, and thereby promote cancer cell proliferation and migration/metastasis, eventually aggravating HCC pathogenesis. As *HAMP*/hepcidin could regulate the activation of this pathway, the authors proposed *HAMP* as a tumour suppressor gene [63]. No wonder, hepcidin decrement in HCC correlates with tumour stage, cancer grade and rapid cancer progression, i.e. cancer aggressiveness [20].

Liver fibrosis and cirrhosis are important risk factors for HCC development [7, 65]. Activated hepatic stellate cells play a very important role in promoting liver fibrosis [15]. Excess iron can affect hepatic stellate cells and mediate/accelerate liver fibrosis via various mechanisms [15, 66]. Hepcidin has shown protective characteristics by suppressing hepatic-stellate-cell activation via inhibition of the TGF-β-induced smad3 phosphorylation [67]. Downregulation of hepcidin in HCC would remove this protective feature offered by hepcidin and exacerbate HCC pathology.

While discussing the effect of hepcidin on HCC pathology, it is worth mentioning the effect of BMPs, the hepcidin inducers. Raised levels of liver BMPs 6 and 4 in HCC patients [6, 30] exert an effect on cancer. Essentially, increased BMP expression and activated BMP-SMAD signalling promotes cancer cell migration and invasion in HCC [6, 68]. BMPs 6 and 4 contribute to tumour progression and carcinogenesis in HCC [68]. Thus, enhanced levels of BMPs in HCC can aggravate HCC pathology. Due to their increased levels in HCC, BMPs could be included in the panel of HCC diagnostic markers.

## Hepcidin–ferroportin axis in HCC

Dysregulated hepcidin–ferroportin axis is linked with increased cancer risk [69]. In HCC, cancerous cells tend to show iron deficiency, high TfR1 levels and low hepcidin expression whereas the adjacent non-cancerous cells show high iron loading, high hepcidin and high ferroportin expression [28], although a study reported that ferroportin mRNA expression did not differ between HCC cancerous and non-cancerous tissues [17]. Nonetheless, this clearly indicates differential regulation of hepcidin in cancerous and non-cancerous cells [28]. Increased hepcidin results in intracellular iron sequestration by the nearby tumour cells and this enhances tumour proliferation. Simultaneously, reduced hepcidin expression in cancer cells in HCC favours iron acquisition by the cancer cells and promotes cancer cell proliferation. As such, the presence of high TfR1 levels in cancer cells would facilitate iron entry into these cells and enhance their proliferation. Thus, decrement of hepatic and serum hepcidin in HCC favours iron availability for tumour cells as this discourages iron sequestration in Kupffer cells and duodenal enterocytes [28].

In HCC, increased ferroportin mRNA expression has shown no correlation with ferroportin protein levels. This could be because of reduced levels of ceruloplasmin in HCC [18, 19, 28]. Under physiological conditions, ceruloplasmin is thought to stabilise ferroportin protein on the cell surface and/or allow the transport of Fe^2+^ via ferroportin into the extracellular environment, thereby supporting ferroportin function [70, 71]. Reduced ceruloplasmin in HCC would prevent its optimal functionality leading to the observed discordance between ferroportin mRNA and protein levels in HCC. Interestingly, ferroportin overexpression can cause cell cycle arrest, and suppress tumour occurrence and growth [69], thereby showcasing the therapeutic potential of targeting the hepcidin–ferroportin axis in cancers.

## Hepcidin in diagnosis, prognosis and therapeutics of HCC

HCC diagnosis is usually based on raised levels of serum alpha-fetoprotein (AFP) and examining the presence of definitive features via imaging techniques. The threshold of the alpha-fetoprotein level of 400 ng/mL has shown better sensitivity and specificity than that of 200 ng/mL [72]. However, AFP-negative HCC also exists and accounts for almost half of the HCC cases. Such cases are difficult to diagnose due to the absence of sufficient reliable biomarkers. In these cases, detection is primarily based on imaging techniques. As AFP-negative HCC could be early-stage and show small tumours, imaging may not be able to provide sufficient sensitivity or specificity. Also, imaging needs sophisticated and expensive equipment. Thus, the diagnosis of AFP-negative HCC poses a challenge in clinical practice. There is a hindrance in the ability to provide early treatment for these patients and offer improved prognosis and patient outcomes [73]. Therefore, there is a need to search for more biomarkers that could be useful for all HCC cases, and specifically for AFP-negative HCC.

Hepcidin exhibits great diagnostic potential for HCC. Earlier, there were challenges around the detection and quantification of hepcidin mRNA from paraffin-embedded tissue sections. These were overcome when *HAMP* mRNA from HCC patients was successfully detected and distinguished from non-cancerous tissue via quantitative PCR and in situ hybridisation method [74]. Another hepcidin-related diagnostic aid could be the existence of a *HAMP* mRNA variant. HCC cell lines showed a significantly raised variant of *HAMP* mRNA (lacking exon 2), which resulted in truncated pre-pro-hepcidin without post-translational cleavage [75]. Quantifying such *HAMP* mRNA variants could serve as diagnostic markers for HCC because the copy number of the *HAMP* mRNA variant was high in the serum exosomes of HCC patients [76].

Hepcidin shows great potential in prognostication too. In alcoholic cirrhosis, patients with lower hepcidin levels showed a higher risk of HCC and overall death [77], thereby presenting hepcidin as a very important HCC risk-factor detector. In HCC patients, hepcidin decrement correlated with cancer aggressiveness and worse ‘overall, disease-specific and relapse-free survival’ [20]. Since reduced hepcidin expression has been linked with poor disease-free status and higher rate of metastasis, downregulated *HAMP* expression has predicted poor outcomes in HCC patients [63].

Modulation of hepcidin expression in HCC would be an interesting therapeutic approach and this would also imply modulation of iron levels in HCC. Deferasirox is an iron chelator. Saeki et al. showed that deferasirox can induce apoptosis and reduce the proliferation of several hepatoma cells lines and suppress liver tumour development in mice models. It can elevate *HAMP* mRNA expression in both tumour and non-tumour tissues in mice. Thus, deferasirox could be used to rescue hepcidin expression and regulate iron homoeostasis in HCC, while it additionally demonstrates tumour-suppressive characteristics. However, this needs further research, particularly in humans because, in the study by Saeki et al., the efficacy of deferasirox could not be ascertained due to dose-limiting toxicities and no responders were observed in their clinical study [78].

If successful, the above approach could be implemented to rescue/increase hepcidin expression in HCC patients that show low hepcidin levels. However, in some HCC cases, hepcidin expression was found to be increased (previously discussed in this review). These cases would require hepcidin to be downregulated. In a mouse model of HCC that showed high liver hepcidin levels, dandelion polysaccharide treatment downregulated the expression of hepcidin and inhibited the IL6-activated JAK-STAT pathway in HepG2 cells. As such, dandelion, the traditional Chinese medicinal herb exhibits anti-inflammatory and antioxidant properties. It can reduce the iron burden in tumours, arrest cell cycle and thereby suppress proliferation of HCC cell line [23]. Essentially, irrespective of whether hepcidin is downregulated or upregulated in HCC, it can be used as a target of modulation to ameliorate HCC pathology.

## Summary

Hepcidin expression is generally downregulated in HCC, despite the presence of high levels of hepcidin inducers, such as iron, BMPs and IL6. This downregulation could be due to the suppression of *HAMP*, TfR2, HJV, ALK2 and/or circular RNA *circ_0004913*, upregulations of matriptase-2 and/or GDF15, and inactivation of *RUNX3* and/or mutations in *TP53*. Regardless, hepcidin downregulation can increase cancer proliferation and exacerbate HCC pathology. Hepcidin could be used as one of the diagnostic markers for HCC. Its downregulation predicts poor patient outcomes presenting hepcidin as a promising prognostic marker. Hepcidin modulation could be a way of amending HCC pathology and enhancing the existing HCC treatment strategies.

## Data Availability

Not applicable.
